# Health equity audits: a systematic review of the effectiveness

**DOI:** 10.1136/bmjopen-2021-053392

**Published:** 2021-11-11

**Authors:** Kim Robin van Daalen, Fiona Davey, Claire Norman, John Alexander Ford

**Affiliations:** 1 Cardiovascular Epidemiology Unit, University of Cambridge Department of Public Health and Primary Care, Cambridge, UK; 2 Department of Public Health and Primary Care, University of Cambridge, Cambridge, UK; 3 Population Health Sciences Insitute, Newcastle University, Newcastle upon Tyne, UK

**Keywords:** public health, clinical audit, epidemiology

## Abstract

**Abstract:**

**Objectives:**

The purpose of this systematic review is to explore whether health equity audits (HEAs) are effective in improving the equity of service provision and reducing health inequalities.

**Design:**

Three databases (Ovid Medline, Embase, Web of Science) and grey literature (Opengrey, Google Scholar) were systematically searched for articles published after 2000, reporting on the effectiveness of HEA. Title and abstracts were screened according to an eligibility criteria to identify studies which included a full audit cycle (eg, initial equity analysis, service changes and review). Data were extracted from studies meeting the eligibility criteria after full text review and risk of bias assessed using the ROBINS-I tool.

**Results:**

The search strategy identified 596 articles. Fifteen records were reviewed in full text and three records were included in final review. An additional HEA report was identified through contact with an author. Three different HEAs were included from one peer-reviewed journal article, two published reports and one unpublished report (n=4 records on n=3 HEAs). This included 102 851 participants and over 148 practices/pharmacies (information was not recorded for all records). One study reviewed health equity impacts of HEA implementation in key indicators for coronary heart disease, type 2 diabetes and chronic obstructive pulmonary disease. Two HEAs explored Stop Smoking Services on programme access and equity. All reported some degree of reduction in health inequalities compared with prior HEA implementation. However, impact of HEA implementation compared with other concurrent programmes and initiatives was unclear. All included studies were judged to have moderate to serious risk of bias.

**Conclusions:**

There is an urgent need to identify effective interventions to address health inequalities. While HEAs are recommended, we only identified limited weak evidence to support their use. More evidence is needed to explore whether HEA implementation can reduce inequalities and which factors are influencing effectiveness.

**Trial registration number:**

The study was registered prior to its conduction in PROSPERO (CRD 42020218642).

**Strengths and limitations of this study**

- This systematic review represents, to our knowledge, the most comprehensive examination of the evidence on the effectiveness of health equity audits used to reduce inequalities in service provision and clinical outcomes.
- A broad, prospectively published rigorous search strategy (registered in PROSPERO)—that included non-English articles and grey literature—was used.
- All included studies were judged to be of moderate or serious risk of bias.
- The study design of the included studies meant that we were unable to assess the impact of concurrent programmes of work.

## Introduction

 The COVID-19 pandemic has exposed and exacerbated structural, longstanding and unjust drivers of health inequalities, including economic disparities, geographical deprivation, occupational risks and systematic racism.^1^ In the UK, the most deprived areas of the country saw a 118% increased death rate from COVID-19 compared with the least deprived.^2^ Likewise, there have been striking inequalities across minority ethnic groups with people from Pakistan and Bangladesh living in the UK having higher death rates in both the first and second waves.^3^ However, the inequalities directly related to COVID-19 are likely to be overshadowed by the inequalities across, for example, socioeconomic, ethnic and gender strata that will indirectly arise from the pandemic’s impact on education, income, welfare, investment, social care and healthcare.^1^ COVID-19 has also compounded existing healthcare inequalities. During 2019–2020, the most deprived decile had 7 % fewer elective admissions than the least, but 51 % more emergency admissions.^4^ While there is now a significant body of data and research describing the problem of health inequalities, there has limited research and data showing what interventions could reduce them and ensure a fair distribution of health resources.

In response to the emerging inequalities related to COVID-19, Public Health England recommended the use of health equity audits (HEA).^5^ HEA is a tool conducted by public health professionals and/or screening providers to measure and address inequalities in the provision of and access to services, related health outcomes and determinants of health between different population groups. They are conducted to address inequalities by providing evidence to show whether local health needs are being met, to identify service delivery practices and to ensure resources are distributed equitably (resources are distributed fairly in relation to need, not necessarily equally). HEAs typically use a sequential audit design in which they collect data on the relevant health and health services outcomes, and inequalities across a range of different factors (eg, socioeconomic differences, area or regional variations, ethnicity, sexuality). The audits are tailored to specific health outcomes/services and are often supplemented with published data on, for example, screening performance. Compared with other countries, the UK has been the predominant implementer of HEAs, although they have also been used in other countries (eg, Canada,^6^ Iran^7^ and Italy).^8^ Examples include an equity audit of the Health Check programme which found lower uptake in men—especially younger men in deprived areas, and those on the learning disability or severe mental illness register.^9^ Furthermore, an equity audit on a diabetic retinopathy screening programme, found that screening was lower in more deprived areas.^10^

HEAs are not a new initiative. In 2002, as part of the UK national health inequalities strategy, HEAs were recommended for all local health systems to address health inequalities. At that time the use of them became widespread, until 2010 when a change in the UK Government led to the cessation of many health inequalities initiatives, in response to the 2008 recession and financial constraint. Their use was further reduced after significant healthcare reforms in England in 2013 .^11 12^ More recently, a number of equity audits have been undertaken in local health systems and their utilisation is likely to continue expanding in response to the COVID-19 pandemic.

Despite the extensive use of HEAs in the past and current recommendations, there is little research on their effectiveness or on the aspects that could make HEAs successful. Therefore, the aim of this study was to assess the effectiveness of HEAs in reducing inequalities and increasing equity, and to explore factors influencing effectiveness. Importantly we focus on studies with a full audit cycle; those that assess existing inequalities, implement changes/interventions to achieve equity and reassess inequalities, rather than those studies which only describe the inequalities and make recommendations.

## Methods

We conducted a systematic review in accordance with established methodology^13^ and reported in line with the Preferred Reporting for Systematic Reviews and Meta-analyses (PRISMA) statement.^14^

### Search strategy and selection criteria

Three electronic databases (Ovid Medline, Embase, Web of Science) and grey literature (Opengrey, Google Scholar) were systematically searched from 2000 to February 2021 drawing on existing inequality and inequity related search terms. Search terms included those related to audits and inequity (eg, equity, access, equality), see online supplemental table 1. We applied forward (a search to find all of the articles that cite back to an article) and backward (a search to find all the cited references in an article) screening of all full-text publications included and relevant publications (eg, reviews and reports). After removing duplicate records, abstracts and titles were double-screened according to the selection criteria by two researchers (KRvD and FD) using the software Rayyan by March 2021. Discrepancies were resolved by a third researcher (JAF). Inclusion criteria were (1) reporting on audits of health equity, (2) focused on health settings, (3) assessing the effectiveness of the audit on reducing health inequalities, (4) any study design and (5) articles in English, Dutch, German, French and Spanish. Studies were excluded if they were (1) published before 2000, (2) solely described the audit protocol and (3) did not assess the effectiveness of the audit, but only the results of the initial inequality assessment. All full texts for studies that satisfied the selection criteria were retrieved and double screened. Any divergences between authors on study eligibility were discussed until consensus was reached.

Data from included studies were independently extracted by two researchers (KRvD and FD). A third researcher resolved any conflicts (CN). The following information was extracted from each study: first author, year of publication, country, aim, study design, data source, population characteristics (eg, size), inequality measures (eg, gender, socioeconomic), health service changes, time of data collection, summary of audit performed and main findings. Terms/categories conflating race and ethnicity are used throughout the paper as a consequence of being commonly used in the HEA and subsequent data collection, but we acknowledge that race and ethnicity are different social concepts. Study authors were contacted for more information where relevant.

### Quality assessment

Two authors (KRvD and FD) independently assessed the quality of individual studies using the Risk of Bias in Non-randomized Studies of Interventions (ROBINS-I) tool, which assess the risk of bias across seven domains (https://www.riskofbias.info/).^15^ Discrepancies between authors were adjudicated by two authors (JAF and CN). Due to the small number of studies it was deemed inappropriate to perform a Grades of Recommendations, Assessment, Development and Evaluation assessment.

### Synthesis

The conduct of meta-analyses or the assessment of publication bias was deemed inappropriate due to the limited number of studies and data heterogeneity. Therefore, the studies were synthesised narratively.

### Patient and public involvement

Due to the nature of the study (systematic review, no patients were involved in conceptualising or conducting the study.

## Results

**Figure 1:**
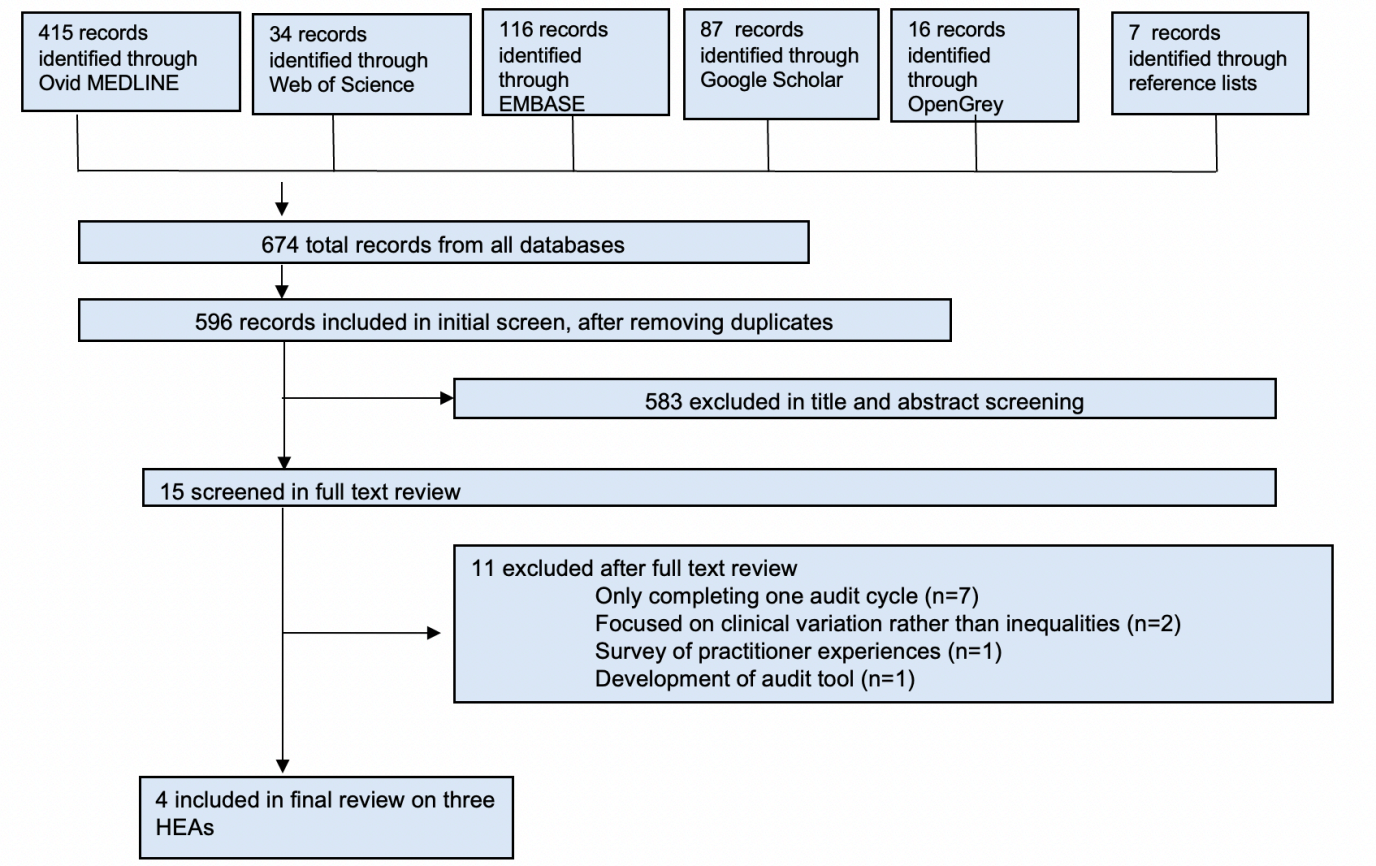
Study selection process. HEAs, health equity audits.

After removal of duplicates our search identified 596 records. Fifteen records were reviewed in full text and three records were included in the final review. An additional follow-up report on a same HEA was identified through contact with an author. This resulted in a total of four included records on three different HEAs. A flow diagram of the screening and selection process can be found in figure 1. We included two HEAs^16^,^18^ reviewing Stop Smoking Services on programme access and equity arising from two published and one unpublished report, and one peer-reviewed intervention study^19^ reviewing health equity impacts of HEA implementation in key indicators for coronary heart disease (CHD), type 2 diabetes mellitus (T2DM) and chronic obstructive pulmonary disease (COPD) (table 1). All included records were conducted in the UK and used a sequential audit design. Across all included HEA there were participants from 148 general practices in London (Newham, City and Hackney, Tower Hamlets) and from general practices and pharmacies participating in the two county Stop Smoking Service programmes in Durham and Lewisham, including a total of 102 851 individuals. Data were collected between 2007 and 2017. The included HEAs assessed various inequalities (including inequalities in ethnicity, gender, age, socioeconomic group and location) in service delivery, service access and health outcomes.^16^,^19^

The majority of published literature on HEAs were one cycle HEA reports that did not assess HEA effectiveness; we identified 56 records which reported only one HEA cycle from grey literature (n=43) and electronic databases (n=13). The majority of these (n=23) were conducted by local governments, local healthcare systems (n=21), or combinations of the former (n=4). A minority were carried out by hospitals (n=3), dental services (n=3) or by national healthcare organisations (n=2). A wide range of services were audited, but the most common were smoking cessation services (n=7), cancer screening (n=7) and health promotion programmes (National Health Service, NHS Health Checks) (n=6).

**Table 1:** Study characteristics

First author, year and country	Aim	Study design	Data sources	Population	Inequality measures (eg, SES, gender)	Resulting recommendations for service delivery changes	Time between data collection
Badrick (2014), UK^19^	The main aim of the study was to describe the development and implementation of practice equity audits, and an evaluation of changing inequalities over time for three project conditions in inner east London.	Sequential audits	Routine clinical and demographic data were collected from practice computer databases, using Morbidity Information Query and Export Syntax software and Web (Egton Medical Information Systems Ltd, 2010) from 148 of the 151 general practices in the three areas of London.	Three areas of London (Newham, City and Hackney and Tower Hamlets) with a combined GP-registered population of 829 710 in mid-2008.	Association between self-reported ethnicity, gender, age-band and four key indicators (cholesterol levels in CHD, blood pressure and haemoglobin A1c levels in diabetes and % smoking in COPD).	38 practices in the intervention arm (Tower Hamlets) received two HEA and facilitated time with a cardiovascular nurse specialist to review their results. The study authors recommended prioritising monitoring inequalities by age, gender, and ethnic group; balancing rigorous, complete reports with simple, brief reports for reaching increased practice audiences; and implementation of HEA facilitation tailored to practice setting and needs to promote changes in clinical performance.	Cross-sectional data were extracted in April of every year between 2007 and 2010 for all patients on the CHD, diabetes and COPD registers.
Pringle (2013), UK^17^	This HEA looks at the use and success of Lewisham’s Stop Smoking Service from April 2007 to March 2012 by age, gender, ethnicity, socioeconomic group and location. In addition, the views of a small number of service users and advisers were sought on factors that may affect the use and success of the service.	Sequential audits	Smoking prevalence data is available from the Integrated Household Survey which combines answers from a no of Office for National Statistics surveys containing questions about smoking. Interviews with 15 smokers and six advisers. Quits dates set from April first 2007 to March 31st 2012 were extracted from Quit Manager. Smoking data is self-reported.	Lewisham residents accessing Stop Smoking services.	Association between age, gender, ethnicity, socioeconomic group, and location and service access rates and successful smoking cessation rates.	The HEA recommended adjusting marketing messages, targeting specific underrepresented groups, collaborating with African American churches to implement Stop Smoking Services, exploring use of innovative technology especially with young smokers, reallocating level three advisers to the underrepresented groups who benefit most from their counselling, and undertaking further research on groups not examined in the HEA.	April 2007 to March 2012.
Roe. (2018), UK^16^Roe (2014), UK^18^	The purpose of these reports is to assess whether the County Durham NHS Stop Smoking Service is having an impact on health inequalities. It aims to identify how services are delivered relative to the deprivation levels across County Durham and provide analysis by the two Clinical Commissioning Groups within its borders. The reports analyse the rate of access and rate of quitters. This HEA also provides a comparison with previous audits conducted in 2007 and 2014.	Sequential audits	Source of the data is Durham County Council Public Health Intelligence Team. The raw data for the 2014 and 2018 HEAs is taken from Quit manager; a Stop Smoking Service web-based patient data management system. The 2007 data was collated from five different reports from localities within Co. Durham and the source of the quit dates is not stated.	2014–Durham residents accessing Stop Smoking services, 23 350 used records2018–Durham residents accessing Stop Smoking Services, 9240 used records	Deprivation was measured at small area level and the Relative Index of Inequality and the Slope Index of Inequality were used to compare inequalities over time.	The HEA recommended targeting specific groups of people including routine and manual workers, Gypsy, Roma and Travellers, pregnant women, people with a diagnosed mental illness, long term conditions and people who live in the 30% most deprived areas.	2014–January 2011 to March 20132018–April 2015 to March 2017

CHD, coronary heart disease; COPD, chronic obstructive pulmonary disease; GP, general practitioner; HEAs, health equity audits; NHS, National Health Service; SES, socioeconomic status.

### HEA implementation

Badrick *et al* implemented and evaluated HEAs in 38 practices in Tower Hamlets Primary Care Trusts (PCTs) which included facilitation sessions encouraging change, identifying areas of expressed difficulty and engaging teams in finding solutions. The intervention tracked four key indicators (blood pressure and haemoglobin A1c levels in DMT2, % smoking in COPD and cholesterol levels in CHD). Changes in performance over time were then examined for the intervention PCTs compared with neighbouring non-intervention PCTs (n=110).^19^ Roe *et al*^16 18^ and Pringle^17^ used a before-and-after comparison rather than the inclusion of a comparison site. Roe *et al*^16 18^ assessed the Durham NHS Stop Smoking Service ’s impact on health inequalities. They explored the rate of access and rate of quitters providing a comparison with audits conducted in 2007, 2014 and 2018 . Slope and Relative Indices of Inequality were calculated by the socioeconomic dimension to inequalities in health.^16 18^ Similarly, Pringle compared differences in access and quitting success rates through the Lewisham NHS Stop Smoking Service between 2000 and 2005 (first HEA) and 2007/2008–2011/2012 (second HEA).^17^

### Changes in inequalities during audit period

All HEAs reported baseline inequitable outcomes in physical health outcomes,^19^ health behaviours and access to or utilisation of health services by age, gender, ethnicity, socioeconomic status and location.^16^,^18^ During the audit period, some degree of reduced inequality was observed in all records compared with the comparison group^19^ or prior HEA data (table 2).^16^,^18^ In Tower Hamlets’ PCTs, reductions in gender and age group differences were found in DMT2 and CHD. Yet, while all ethnic groups showed improvement over the years of HEA implementation, there was no reduction in difference between ethnic groups. Furthermore, some groups showed a widening of inequalities. For example, in the CHD register South Asians increased from being 1.9 (1.6–2.2) times more likely than White groups to have cholesterol levels <94 mmol/L in 2007 to being to 2.4 (2.0–2.8) more likely in 2010.

**Table 2:** Study results

First author, year	Summary of audit	Main findings
Badrick (2014), UK^19^	The audit aimed to reduce health inequalities by ethnicity, age and gender in the management of three common chronic diseases (CHD, DMT2 and COPD).	Baseline inequalities in each condition across the three east London areas were identified. At a crude level, performance in cholesterol, BP and HbA1c improved in all areas over time. All ethnic groups showed improvement, but there was no evidence of a reduction in differences between ethnic groups.Over the 3 year study, a reduction in health inequalities was measured in some groups (such as patients over 85 years with diabetes) with only slight reductions in, continued, or worsened inequalities observed in most other groups. Compared with the neighbouring areas, Tower Hamlets (receiving the intervention) had smaller improvement levels in CHD, higher absolute changes in both diabetes measures, and small but similar changes in rates of smoking in COPD patients. The study reported positive GP responses to the intervention providing assistance in conducting/interpreting HEAs.Reductions in gender and age group differences were noted in DMT2 and CHD.
Pringle (2013), UK^17^	This HEA looks at the use and success of Lewisham’s SSS from April 2007 to March 2012 by age, gender, ethnicity, socioeconomic group and location. In addition, the views of a small no of service users and advisers were sought on factors that may affect the use and success of the service.	Since the last equity audit more smokers from ‘black and ethnic minority groups’ were using the service. In addition, this HEA shows that over the last 5 years the SSS was reaching an increasing number of people from deprived areas. More quit dates were set by smokers from deprived areas than from less deprived areas. Overall, this HEA shows inequality across Lewisham’s smokers in the use and success of Lewisham’s SSS in terms of the need for SSS. The population groups that seemed to be underrepresented in their use of the service were: younger smokers, older women, Indian men, Chinese men, white Irish men and black African smokers. Additionally, smokers from more deprived areas, routine and manual workers, students and unemployed smokers were less likely to successfully quit smoking.
Roe (2014), UK^16^Roe (2018), UK^18^	This HEA assesses the distribution of the Durham SSS and its effectiveness relative to deprivation levels within County Durham and the two clinical commissioning groups within its borders.	2014—Compared with the results of the 2007 HEA there has been an increase in the relative index of inequality for access and quit rates as well as a reduction in the difference between the two, indicating that the County Durham SSS is contributing to a reduction in health inequalities.2018—The County Durham SSS has been successful in contributing to a reduction in the equity gap, seeing a consistent increase in the relative index of inequality for access and quit rates. This was true for services accessed in pharmacies, primary care, and specialist SSS. The audit found a higher rate of pregnant smokers in more deprived areas, but also a higher quit rate for pregnant smokers who accessed the services in more deprived areas. This indicates that the County Durham SSS is contributing to a reduction in health inequalities.

BP, blood pressure; CCG, Clinical Commissioning Groups; CHD, coronary heart disease; COPD, chronic obstructive pulmonary disease; DMT2, type 2 diabetes mellitus; GP, general practitioner; HbA1c, haemoglobin A1c; HEA, health equity audit; SSS, Stop Smoking Service.

Similarly, smoking rates in COPD indicate increased disparity between white and other ethnic groups in 2010.^19^ The audit of the Lewisham Stop Smoking Service found an increase in service access by ‘black and ethnic minority groups’ as well as by people from deprived areas (2007/2008–2011/2012) as compared with prior audits (2000–2005). However, the HEA report also identified several population groups still under-represented in access rates (eg, younger smokers, older women, Indian men, Chinese men, white Irish men and black African smokers) and overall inequality in programme access and success rates.^17^ The 2014 Stop Smoking Service HEA in County Durham found a reduction in health inequalities compared with prior audits (2007) as demonstrated by a consistent increase in the relative index of inequality, the size of the gap between the least and the most deprived areas expressed as the average rate over all wards, for access and smoking quit rates. Furthermore, a reduction in access rate to quit rate was observed—gap of 69%–16% in 2007 and 2014, respectively.^16^ Reductions in the inequality gaps were observed again in the 2018 HEA compared with the 2007 and 2014 HEAs.^18^

### Study quality assessment

Study quality assessment was conducted using the ROBINS-I tool. Each included record was found to have a serious or moderate risk of bias in the various categories assessed (table 3). Confounding may have influenced the results of the reports due to the inadequacy of study designs to differentiate effectiveness of HEA implementation from simultaneously implemented local improvement initiatives, the ‘noise’ of a changing NHS or other societal changes that may have led to reduced or increased inequalities. The potential selection of health practices that already established an equity-focus may have resulted in selection bias, meaning that results may not be generalisable to most areas in the UK. Likewise, as included studies have solely been performed in the UK results may not be applicable to other countries.

**Table 3:** Risk of bias—ROBINS-I tool

Study	Bias due to confounding	Bias due to selection of organisations into study	Bias in classification of interventions	Bias due to deviations from intended interventions	Bias due to missing data	Bias in measurement of outcomes	Bias in selection of reported result
Badrick *et al* (2014), UK^19^	Serious	Serious	Low	No information	Low	Moderate	Low
Pringle (2013), UK^17^	Serious	Serious	Low	No information	Moderate	Moderate	Moderate
Roe and Woodall (2014), UKRoe (2018), UK^16 18^	Serious	Serious	Low	No information	Moderate	Moderate	Low

## Discussion

This systematic review represents, to our knowledge, the most comprehensive examination of the evidence on the effectiveness of HEA. We identified three HEAs^16^,^19^ based in healthcare or public health settings with serious to moderate risk of bias. All showed the presence of baseline inequalities and found reductions in health inequalities across various strata in the subsequent years of initial HEAs. Only one study used comparison sites.^19^

### Meaning of the results

There has been little research undertaken to explore the effectiveness of HEAs, despite them being widely used in the UK during the 2000 s and currently being recommended by PHE.^5 11^ This may be because of methodological challenges in assessing effectiveness or an assumption that they are the right strategy. The majority of HEAs we identified only undertook one cycle, suggesting that practitioners tend to use HEAs as a tool to assess the existing inequalities within a service rather than a tool to record or reduce inequalities over time. Although HEAs may be useful at identifying areas of health inequality or greater need, without repeating the data collection it is not possible to say whether the HEA resulted in any meaningful service change or targeted intervention, let alone whether this resulted in a reduction in inequities.

We only identified three HEAs that completed the audit cycle to assess if the recommendations and changes resulted in a reduction in inequalities over time. The lack of peer-reviewed research assessing HEA effectiveness may reflect the lack of healthcare and public health services to evaluate the impact and effectiveness of decisions, with a much greater focus on addressing problems. It may also reflect difficulties presented by frequently changing priorities and frequent turnover of staff. Furthermore, a reluctance to publish HEAs may be present as they could cause reputational damage to organisations or even a concern that the findings may leave the organisation open to legal challenge under equality legislation. Qualitative research around clinical audit has shown that audit is seen as ‘ka time-consuming, additional chore and a managerially driven exercise’^20^ that is hampered by a lack of resources, lack of expertise, lack of audit plan, and organisational impediments.^21^ Organisational change and austerity measures have meant that local authority Public Health teams have faced increased responsibilities and real-terms funding cuts.^22^

The single peer-reviewed article was undertaken in a number of general practices in London. The authors found that it was possible to undertake equity audits in general practice using routine data. While all of the included studies identified some reductions in health inequalities during the HEA process, only Badrick *et al* had a suitable comparison group. Furthermore, in the absence of randomised intervention studies and further high-quality observational studies, attributing changes in equities to HEAs based on the included reports is inappropriate due to the potential confounding and biases introduced. No evidence was found to suggest that HEAs result in harm or should not be undertaken in the absence of further evidence.

### Comparison with existing literature

Aspinall and Jacobson^23^ undertook a baseline survey in 2004 of practitioners’ experiences across England in the first HEA implementation year of undertaking nationally mandated. The authors found that national target-setting, national guidance on self-assessment and the inclusion of HEAs within a ratings system influenced whether the process and, in a significant minority, implementation of the findings of HEAs became part of healthcare systems’ routine business.^23^

There is a sizeable body of research looking at the effectiveness of clinical audits (ie, non-equity focused). For example, a Cochrane review examined the impact of audit and feedback on professional behaviour. The authors identified 140 randomised controlled trials and found that audit and feedback has small but important improvements in professionals behaviour.^24^ Similarly, there is evidence for the use of quality improvement methods with some consideration to equity. Lu *et al* found that about a third of quality improvement projects in diabetes care included an equity perspective.^25^

However, these findings are not necessarily transferable to HEAs as clinical audits and quality improvement programmes are generally undertaken at a smaller organisation level and focus on adherence to evidence-based best practice guidance. HEAs are generally implemented at a higher organisational level, such as across a local government level or healthcare system, and it is not always clear what actions are needed to reduce the inequalities gap. To illustrate, Regmi and Mudyarabikwa undertook a review of factors that support the reduction of inequalities in local healthcare systems in the UK and found that there was little evidence that local healthcare arrangements alone were effective in reducing health inequalities.^26^

However, there are a number of principles drawn from clinical audit and quality improvement methods which may be effective in HEAs. Grimshaw *et al* argue for an implementation laboratory where there is a continual cycle of testing different interventions and implementation strategies through audit and feedback which may be effective in reducing health inequalities through HEA implementation.^27^

### Strengths and limitations

Our research used a prospectively published rigorous systematic review strategy that included non-English articles and grey literature. We had a robust process for screening titles/abstracts and full texts, extracting data and determining the risk of bias using a validated tool for quality assessment. However, only one HEA with multiple years of data was found in the peer reviewed literature and all reports included were of low to moderate quality. It is likely that there are a number of HEAs not in the public domain. Yet, based on our research, most of these are likely to only contain one HEA cycle. There may be a publication bias towards studies reporting positive results (ie, reductions in inequalities). Importantly, as the reports included are sequential audits rather than well-designed randomised studies, they may not have been equipped to differentiate HEA effectiveness from simultaneously implemented local improvement initiatives, the ‘noise’ of a changing NHS or other societal changes that may have led to reduced or increased inequalities.

### Implications for research and policy

While the efforts to address inequalities in healthcare are not new, the impacts of the pandemic have starkly delineated the imperative to do so. There is an urgent need to find effective interventions to reduce health inequalities. Public Health England recommends the use of HEAs and has published accompanying guidance describing step-by-step processes of HEA implementation.^5^ Yet, thus far, it is unclear whether this has been supported by scientific evidence. It is likely that there are key factors that will make HEA undertaking effective in inequality reduction and factors that will not. For example, previous research has found that audits tend to be more effective when feedback is given by respected colleagues, there is frequent data presentation, both goals and action-plans are included and the recipients are non-physicians.^28^ Therefore, further well-designed studies with suitable comparison groups are essential to further inform on the effectiveness of HEAs. Process evaluation is needed to understand the factors that optimise HEA effectiveness and implementation processes. Decision-makers may be more likely to change behaviour based on case examples of how HEAs have been used to reduce inequalities.

While there is limited evidence for use of HEAs, we do believe that they should still be used until further research is undertaken because we did not find any evidence of harm and there is a logical rationale by which they could reduce inequalities. The priority for policy-makers is evaluating ongoing HEA and generating the evidence base to understand if they work and, if so, what makes them most effective.

## Conclusion

Research and practice demonstrate that meaningfully impacting inequalities in both health outcomes and healthcare delivery is a complicated, challenging task faced by already overburdened and under-resourced health systems. While HEA implementation is currently recommended, evidence for their effectiveness in reducing inequalities is sparse. This evidence gap requires action. Efforts to reduce inequalities must neither be avoided nor delayed because of their complicated nature; nor should they be undertaken haphazardly without much needed, evidence-based guidelines. Further research is needed to assess their effectiveness and understand what makes them effective (or not).

## Supplementary material

10.1136/bmjopen-2021-053392 online supplemental file 1

## Data Availability

No data are available.
