# Supplementary material for: Health equity audits: a systematic review of the effectiveness
Source: BMJ Open. 2021 Nov 11;11(11):e053392. doi: 10.1136/bmjopen-2021-053392 (PMC8587574; doi:10.1136/bmjopen-2021-053392)
Supplement: online supplemental file 1 [file bmjopen-2021-053392supp001.pdf]

Supplement Material to Systematic Review on the Effectiveness of Health Equity Audits: existing evidence and call for further research.

Supplementary Table 1: Search Strategy

Database	Search Strategy
Ovid MEDLINE(R) and Epub Ahead of Print, In-Process & Other Non-Indexed Citations, Daily and Versions(R)	(Audit*).ti. or exp *management audit/ or exp *clinical audit/<br>AND<br>(Health* adj3 (equit* or inequit* or equalit* or inequalit* or disparit* or access* or inaccess*)).ti. or (socioeconomic).ti. or exp *health services accessibility/ or exp *socioeconomic factors/ or exp *health status disparities/ or exp *healthcare disparities/
Embase	(Audit*).ti. or exp *management/ or exp *clinical audit/<br>AND<br>(Health* adj3 (equit* or inequit* or equalit* or inequalit* or disparit* or access* or inaccess*)).ti. or (socioeconomic).ti. or exp *health services access/ or exp *socioeconomics/ or exp *health disparity/
Web of Science	TI=(Audit*)<br>AND<br>TI=(Health* near/3 (equit* or inequit* or equalit* or inequalit* or disparit* or access* or inaccess*)) or TI=(socioeconomic)
OpenGrey	Health* equi* audit<br>health* inequali* audit<br>health* disparit* audit<br>health* inaccessibility audit<br>health* accessibility audit
Google	allintitle: audit "healthcare inequalities" filetype:pdf<br>allintitle: audit "healthcare disparities" filetype:pdf<br>allintitle: audit "healthcare inaccessibility" filetype:pdf<br>allintitle: audit "health equity" filetype:pdf<br>allintitle: audit "health inequalities" filetype:pdf
