# Supplementary material for: Health equity audits: a systematic review of the effectiveness
Source: BMJ Open. 2021 Nov 11;11(11):e053392. doi: 10.1136/bmjopen-2021-053392 (PMC8587574; doi:10.1136/bmjopen-2021-053392)
Supplement: Supplementary file 2 [file bmjopen-2021-053392.reviewer_comments.pdf]

PEER REVIEW HISTORY

BMJ Open publishes all reviews undertaken for accepted manuscripts. Reviewers are asked to complete a checklist review form (**http://bmjopen.bmj.com/site/about/resources/checklist.pdf**) and are provided with free text boxes to elaborate on their assessment. These free text comments are reproduced below.

ARTICLE DETAILS

**TITLE (PROVISIONAL)**	Health Equity Audits: a systematic review of the effectiveness
**AUTHORS**	van Daalen, Kim; Davey, Fiona; Norman, Claire; Ford, John

VERSION 1 – REVIEW

**REVIEWER**	Peter Aspinall<br>University of Kent, Centre for Health Services Studies
**REVIEW RETURNED**	27-Jul-2021

**GENERAL COMMENTS**	This nicely written paper addresses a relatively neglected topic, that is, the effectiveness of health equity audits through their full set of cycles. This is a timely paper given that Public Health England has recommended the use of health equity audits following the coronavirus pandemic.<br>The systematic review uses robust methods though perhaps more could have been said in the ‘methods section’ about how some of the sources were used, such as the Web of Knowledge Citation Indices and the use of article reference lists. Also, it was a little surprising to see that SCOPUS was not cited as a database source.<br>I do not have any major criticisms of the paper which would make a very useful contribution to the published evidence base on this topic and is likely to attract a high citation rate.<br>There are, however, a number of minor infelicities that need addressing.<br>p. 8, lines 15-20: ‘terms/categories conflating race and ethnicity….’ – ‘race’ is little used in government designation and data collection – see, for example, ‘Ethnicity Facts & Figures’ (race disparity audit)<br>p. 8, line 46: conduction – conduct?<br>p. 10, line 39: - by through?<br>p. 11, line 28 (table): Office of National Statistics should be Office for National Statistics<br>p. 12, line 10: GRT - commas needed between component groups<br>p. 13, line 19: al – all?<br>p. 13, line 39 – ‘black and minority ethnic groups’ – this construction has been criticised as ‘black groups’ are ‘minority ethnic groups, though if used in the cited paper should be retained, perhaps in quote marks (the same applies to ‘black and minority ethnic groups’ on p. 15, line 16).<br>p. 17, line 26 – may exists?<br>p. 20, line 41 – process evaluation is needed<br>The database ‘Google Scholar’ is cited on p. 3 and p. 7 but ‘Google’ on p. 26 and p. 27. They are different databases.

**REVIEWER**	Janya McCalman<br>Central Queensland University, Health, Medical and Applied Science
**REVIEW RETURNED**	02-Aug-2021

**GENERAL COMMENTS**	Thank you for providing this interesting and informative paper.

	My main concern is about the confusion throughout the paper regarding concepts of equity and equality. The term "equity" - (i.e. recognition that each person has different circumstances and allocation of different resources and opportunities needed to reach an equal outcome) is used throughout despite the review of research that assesses equality of healthcare and health outcomes across racial/ethnic groups. A clarification, justification and/or change of the terms used is needed.<br>Abstract results would benefit from clarity about the jump from the 596 articles to the 3 HEAs (in terms of the selection process of publications and representation of HEAs within publications). Also the PRSMA diagram records 4 publications.<br>Introduction - page 4 lines 19 + - It would be useful to the international reader to have a more detailed description of what information is requested for a HEA? Do they use routinely collected data? Who conducts the audits?<br>Methods - please explain forwards and backwards screening.<br>Minor typos are indicated in the attached file.<br><br>The reviewer provided a marked copy with additional comments. Please contact the publisher for full details.

VERSION 1 – AUTHOR RESPONSE

Reviewer: 1
Mr. Peter Aspinall, University of Kent
Comments to the Author:
This nicely written paper addresses a relatively neglected topic, that is, the effectiveness of health equity audits through their full set of cycles. This is a timely paper given that Public Health England has recommended the use of health equity audits following the coronavirus pandemic.

Thank you for these positive comments.

The systematic review uses robust methods though perhaps more could have been said in the 'methods section' about how some of the sources were used, such as the Web of Knowledge Citation Indices and the use of article reference lists. Also, it was a little surprising to see that SCOPUS was not cited as a database source.

Based on an initial scoping review we knew that the majority of the studies would be covered in health care literature covered by MEDLINE and EMBASE. We included web of science to broaden the search and a broad grey literature search to ensure we didn't miss any key literature. We decided not to include SCOPUS because we were reasonably confident that the other methods would have identified the key literature.

I do not have any major criticisms of the paper which would make a very useful contribution to the published evidence base on this topic and is likely to attract a high citation rate.
There are, however, a number of minor infelicities that need addressing.
p. 8, lines 15-20: 'terms/categories conflating race and ethnicity….' – 'race' is little used in government designation and data collection – see, for example, 'Ethnicity Facts & Figures' (race disparity audit)

We thank the reviewer for this feedback and have adapted the sentence accordingly.

p. 8, line 46: conduction – conduct?

This has been adopted accordingly.

p. 10, line 39: - by through?

“By” has been removed from the sentence.

p. 11, line 28 (table): Office of National Statistics should be Office for National Statistics

This has been adopted accordingly.

p. 12, line 10: GRT - commas needed between component groups

This has been adopted accordingly.

p. 13, line 19: al – all?

This has been adopted accordingly.

p. 13, line 39 – ‘black and minority ethnic groups’ – this construction has been criticised as ‘black groups’ are ‘minority ethnic groups, though if used in the cited paper should be retained, perhaps in quote marks (the same applies to ‘black and minority ethnic groups’ on p. 15, line 16).

As it is cited from the paper we have changed it to ‘black and minority ethnic groups’.

p. 17, line 26 – may exists?

This has been changed to ‘may be present’.

p. 20, line 41 – process evaluation is needed.

This has been adopted accordingly.

The database ‘Google Scholar’ is cited on p. 3 and p. 7 but ‘Google’ on p. 26 and p. 27. They are different databases.

The figure has been adopted in order to read “Google Scholar”.

Reviewer: 2
Dr. Janya McCalman, Central Queensland University
Comments to the Author:
Thank you for providing this interesting and informative paper.

My main concern is about the confusion throughout the paper regarding concepts of equity and equality. The term "equity" - (i.e. recognition that each person has different circumstances and allocation of different resources and opportunities needed to reach an equal outcome) is used throughout despite the review of research that assesses equality of healthcare and health outcomes across racial/ethnic groups. A clarification, justification and/or change of the terms used is needed.
Thank you for the feedback. In our paper we have tried to align the language used with the language used by the NHS. In their description of HEAs the NHS describes them as:

“To address inequalities, we should distribute resources fairly in relation to need, not necessarily equally, as shown in the illustration above. We can use health equity audits (HEAs) to identify health inequalities between different population groups.”
Hence we decided to apply the words “inequality” and “inequity” in the same way throughout the paper.
Abstract results would benefit from clarity about the jump from the 596 articles to the 3 HEAs (in terms of the selection process of publications and representation of HEAs within publications).

We fully agree with the reviewer’s comment and have adapted the abstract accordingly. See page 2, lines 13-17.

Also the PRSMA diagram records 4 publications.

This is indeed correct. There are 4 records included in the paper, however they discuss only three HEAs. Hence we have worded it as “Fifteen records were reviewed in full text and three records were included in the final review. An additional follow-up report on a same HEA was identified through contact with an author. This resulted in a total of four included records on three different HEAs. A flow diagram of the screening and selection process can be found in figure 1. We included two HEAs15,17,18 reviewing Stop Smoking Services on program access and equity arising from two published and one unpublished report, and one peer-reviewed intervention study19 reviewing health equity impacts of HEA implementation in key indicators for coronary heart disease (CHD), type 2 diabetes (DMT2), and chronic obstructive pulmonary disease (COPD) (Table 1).” in the results section. We have also tried to make this clear in the flow diagram.

See page 8, lines 11-15 and page 10.

Introduction - page 4 lines 19 + - It would be useful to the international reader to have a more detailed description of what information is requested for a HEA? Do they use routinely collected data? Who conducts the audits?

We agree with the reviewer’s feedback and have provided more detail on the HEAs in the introduction. See page 4 line 24-26 and page 5 line 1-7.

Methods - please explain forwards and backwards screening.

We agree with the reviewer that this needed explanation and have added the definition between brackets as follows:

“We applied forward (a search to find all of the articles that cite back to an article) and backward (a search to find all the cited references in an article) screening of all full text publications included and relevant publications (e.g. reviews and reports).”

See page 6, line 19-21.

Minor typos are indicated in the attached file.

All typos have been corrected accordingly.
